# Feeding-Related Early Signs of Autism Spectrum Disorder: A Narrative Review

**DOI:** 10.3390/jpm14080823

**Published:** 2024-08-02

**Authors:** Kamila Castro, Richard E Frye, Eduarda Silva, Cristiane Vasconcelos, Laura Hoffmann, Rudimar Riesgo, Juliana Vaz

**Affiliations:** 1 Serviço de Neuropediatria do Hospital de Clínicas de Porto Alegre, Porto Alegre 90035-903, RS, Brazil; rriesgo@hcpa.edu.br; 2 Programa de Pós Graduação em Saúde da Criança e do Adolescente, Universidade Federal do Rio Grande do Sul, Porto Alegre 90610-000, RS, Brazil; cristianepereira@hcpa.edu.br; 3 Programa de Pós-Graduação em Nutrição e Alimentos, Universidade Federal de Pelotas, Pelotas 96010-610, RS, Brazil; eduarda.silva@ufpel.edu.br (E.S.); laura.hoffmann@ufpel.edu.br (L.H.); juliana.vaz@ufpel.edu.br (J.V.); 4 Autism Discovery and Treatment Foundation and Rossignol Medical Center, 4045 E Union Hills Rd, Phoenix, AZ 85050, USA; drfrye@autismdiscovery.org; 5 Faculdade de Nutrição, Universidade Federal de Pelotas, Pelotas 96010-610, RS, Brazil

**Keywords:** autism spectrum disorder, child development, eating, feeding behavior, satiety, review

## Abstract

Feeding difficulties are prevalent among individuals with autism spectrum disorder (ASD). Nevertheless, the knowledge about the association between feeding-related early signs and child development remains limited. This review aimed to describe the signs and symptoms related to feeding during child development and to explore their relevance to the diagnosis of ASD. Specialists in nutrition and/or ASD conducted a search of MEDLINE, PsycINFO, and Web of Science databases. Although studies in typically developing children demonstrate age-related variations in hunger and satiety cues, the literature about early feeding indicators in ASD is scarce. Challenges such as shortened breastfeeding duration, difficulties in introducing solid foods, and atypical mealtime behaviors are frequently observed in children with ASD. The eating difficulties experienced during childhood raise concerns for caregivers who base their feeding practices on their perceptions of food acceptance or refusal. Considering the observed associations between feeding difficulties and ASD, the importance of recognizing feeding-related signs according to developmental milestones is emphasized to alert medical professionals that deviation in the formation of feeding habits and skills could indicate the need for ASD diagnostic investigation.

## 1. Introduction

Neurodevelopment is, unsurprisingly, extremely complex and involves multiple processes including neurulation, neuronal proliferation and migration, apoptosis, synaptogenesis, and myelination [1]. Early childhood is a critical period of brain growth that coincides with the emergence of nearly all cognitive, behavioral, and social/emotional functions. The brain is particularly susceptible to postnatal growth deficits, and brain areas such as the cerebellum and cortical gray matter exhibit the highest growth rates, with a four- to five-fold increase in volume. Moreover, the cortex undergoes a remarkable transformation from a largely lissencephalic structure at the end of the second trimester to a highly convoluted mantle at around full-term gestation that approximates the morphology of the adult cerebral cortex [2]. Critical development windows exist for the different brain regions, including those linked to high-order functions [3]. 

Nutrient intake during early postnatal growth influences brain growth and maturation with subsequent effects that persist into childhood and adolescence [4]. Notably, recent studies indicate that early postnatal development depends on the nutritional status during the prenatal period [5], suggesting that inadequate nutrition during critical periods of development is more likely to lead to permanent rather than transient effects on the brain [6]. For example, studies have shown that deficiencies in key nutrients such as zinc and vitamin D put offspring at risk for neurodevelopmental disorders such as autism spectrum disorder (ASD) [7].

According to the Diagnostic and Statistical Manual of Mental Disorders, Fifth Edition (DSM-5-TR), Feeding and Eating Disorders are defined as persistent disturbances in feeding and feeding-related behaviors that result in the altered consumption or absorption of food and that significantly impair physical health or psychosocial functioning [8]. “Feeding difficulties” is an umbrella term that has been used to describe problems with limited food intake, restrictive diets, and the impact on nutrition, and food preference [9,10]. Currently, the literature on ASD and factors related to food is growing [11,12,13]. However, few studies provide longitudinal evidence or methods that are robust enough to have effective answers about these aspects in these patients. Children with ASD have a higher risk of feeding problems [14], such as food selectivity, and many of these feeding difficulties continue into childhood, persist in adolescence, and even spill over into adulthood [15]. These symptoms, sometimes, are most distressing to the child’s family and healthcare professionals as they impact the child’s adaptive function and health [16]. Additionally, the lack of food variety may put individuals at risk for nutritional inadequacy and brain development deficits. 

It is important to highlight that feeding difficulties in patients with ASD could originate from different aspects, such as behavioral or sensorial dysfunctions. Some feeding difficulties can manifest themselves in the first months of life and overlap with the symptoms of ASD, potentially being an important warning sign which could assist with an accurate, early diagnosis and an individualized intervention. It is important to highlight that there is no clear description in the literature of the signs of hunger and satiety during child development. Although there are developmental milestones which are crucial in terms of monitoring the development of babies and children [17,18], they do not present points related to nutritional aspects. Combining this argument with the fact that there is a large number of ASD patients with feeding difficulties, the main focus of this review was to investigate the main characteristics (or lack of them) related to hunger and satiety that can be considered warning signs of ASD during development, and critically analyze how these aspects can aid in the early diagnosis.

## 2. Materials and Methods

This review followed the methodology described by Mak et al. [19]. After identifying a team of experts on nutrition and/or ASD, the next step was to define the research question. This review will investigate if the signs of hunger and satiety (or their absence) during child development can be used as a warning sign for investigating ASD. 

A non-systematic review of MEDLINE, PsycINFO, and Web of Science databases was conducted. The search focused on original studies and case reports. No limits were applied in terms of the language or year of publication. All the studies that presented information concerning manifestations related to hunger and satiety during child development were included. Specifically on ASD, the studies that provided information about behavior during meals/food were included. Additionally, references from the papers included were also verified.

A series of meetings were held in order to discuss and extract data from the papers in a consistent manner. The data extracted from the papers included sample information (sample size, age, and sex), methodological aspects, and main findings of the studies. This process was conducted independently by all the authors. A critical analysis of the topic was carried out so that all the points could be discussed. Additionally, a stakeholder was consulted in order to provide insights on the topic and improve the review.

## 3. Results

### 3.1. What Are the Expected Nutritional Skills during Typical Development?

In order to know what can actually be considered atypical nutritional development, typical nutritional development (TD) must be known in detail. Currently, the development milestones involve motor and social skills and do not include items related to eating [17,18]. It is important to highlight that nutritional skills are learned from early childhood and that they need to be stimulated. Table 1 describes important TD nutritional skills and the warning signals of the atypical development of these skills.

Appetite is a complex trait that is susceptible to changes through childhood according to different factors, including environmental influences, such as gestational weight gain and mode of feeding, as well as cognitive development [20,21,22]. A longitudinal study, which applied the Baby Eating Behavior Questionnaire at 3 months (n = 347) and the Children’s Eating Behavior Questionnaire for toddlers at 12 months of age (n = 325), found significant changes in appetite traits with increasing age, suggesting limited stability in these characteristics [20]. A longitudinal study (n = 38) which analyzed video records of meals during four different developmental periods (6 months, 6–12 months, 12–18 months, and 18–24 months) revealed a significant change in behavioral interaction during meals over time. The signs of engagement, such as verbal cueing and mutual gaze, increased with age; however, disengagement cues were not affected by follow-up time [23].

A cohort study conducted in the United States (n = 127) which applied the Children’s Eating Behavior Questionnaire to children aged 2 to 15 years found that satiety responsiveness declined with age while emotional overeating increased. Food fussiness tended to increase until six years of age, followed by a decline [24]. These findings are in line with another review that suggested a decrease in the self-regulation of hunger and satiety according to age [25].

Hodges et al. [26] performed an observational cohort study (n = 45) that assessed hunger and fullness cues in mother/infant dyads from 3 to 18 months through the Responsiveness to Child Feeding Cues Scale and video records analysis. Between three and six months of age, the signs of interest in food included opening the mouth wide and settling into feeding, while postural attention and active searching for food became evident between six and eighteen months of age. Satiation cues identified during the first six months of life included a decrease in activity levels and muscle tone, while after six months of age, behaviors such as playing with food, pushing food away, and using verbal or non-verbal negative expressions were seen. These findings were supported by a systematic review, which also reported the wish to eat less often during the day, pulling food away, and spitting food out as fullness cues [22].

The literature presents a series of questionnaires and protocols that assess the difficulties linked with nutrition at different ages; however, few are specific to ASD. Among so many specifications for this group of patients, the questionnaires for children with typical development often fail to correctly reflect the feeding difficulties or current nutritional demands of each patient. Table 2 presents a list of questionnaires to assess feeding difficulties from the studies included in this review.

During the development of the self-regulation of hunger and satiety, it is believed that children initially engage in an implicit and automatic process of eating in response to external stimuli. As a result, it subsequently leads to the explicit processing of these signs related to eating [49]. Therefore, alterations in this process may have an impact on the construction of eating behavior. Individuals with ASD often experience challenges in processing social information, affecting behavioral response and reciprocity [49]. In addition, sensory processing difficulties are commonly observed in these patients, potentially interfering in interoception and leading to an altered perception of hunger and satiety [16].

Food refusal and limited variety are frequently observed in TD children as a result of the physiological aspects of development and social influences [50,51]. In a population-based longitudinal study (n = 3748) that evaluated the trajectory of picky eaters between one and six years of age, approximately a third of the children were remitting picky eaters, with their condition improving before six years of age. However, the persistence of this characteristic throughout the entire period of evaluation was found in about 5% of the participants and was significantly associated with pervasive developmental problems [52] This finding may serve as an important indicator for evaluation.

### 3.2. Developmental Feeding Skills in Patients with ASD from Birth to 1 Year Old

According to a recent review, individuals with ASD have a higher prevalence of oral motor skill deficits affecting essential functions in the feeding process, such as chewing and swallowing [53]. Motor impairments may lead to difficulties in breastfeeding, resulting in the need for infant formula. For instance, a retrospective cross-sectional investigation of medical records (n = 105) identified motor problems as an early sign of ASD diagnosis, including the absence of sucking reflex and hypotonia in the first years of age. Furthermore, the same study identified scarce and selective feeding as the early signs of ASD, present in 11.4% and 25.3% of the sample, respectively [54]. Motor anticipation failure during feeding has also been demonstrated in a retrospective analysis of family home movies of 27 infants between the ages of three and six months [55].

A meta-analysis demonstrated that children diagnosed with ASD are significantly less likely to be exclusively or partially breastfed when compared to TD children [56]. Accordingly, difficulties in breastfeeding were reported by 47% of the mothers of 18 children with ASD, compared with 20% of a group of 20 TD children, in a cross-sectional study of retrospective data [57]. Higher ASD traits at six years of age were significantly associated with the use of infant formula and behaviors related to appetite regulation, such as drinking in small amounts and appearing hungry or unsatiated at two months of age, in a population-based cohort that included 3546 children [58]. Comparably, there is also evidence in the literature of the dysregulation of food intake and appetite in individuals with ASD [59].

Feeding difficulties may initially manifest not only as issues with breastfeeding but also as challenges in introducing solid foods. These findings are supported by a longitudinal study of 4930 individuals aged 1.5 to 14 years of age which reported that eating problems are frequently correlated with ASD symptoms and may persist from early childhood into adolescence [60]. Children with ASD may have a late introduction of complementary feeding when compared to TD controls according to a cross-sectional study [61]. Similarly, the analysis of data on feeding and food frequency from 6 to 54 months of age identified that children with ASD (n = 79) had a delayed introduction of solids after six months of life [14]. Parents commonly report intolerance to specific textures, more attempts required to introduce new foods, and a shortened time of breastfeeding during the first year of life [62]. These reports could be considered as the early signs of sensory processing difficulties, which are prevalent in ASD and considerably affect eating behavior [63]. 

### 3.3. Developmental Feeding Skills in Patients with ASD from 1 to 3 Years Old

The literature reports that eating difficulties persist beyond infancy in patients with ASD. Although organic inappetence may occur around the age of two years, their food consumption moderates to match a slower rate of growth [64]. In patients with ASD, there is often no return to usual consumption after this period. The eating behavior of young children with ASD is often a cause of concern for parents and one of their most frustrating difficulties. This is probably because they are likely to refuse food and demand specific utensils and food presentations [57]. Retrospective data, derived from mothers’ reports, revealed a correlation between the age and an increase in feeding-related concerns in children with ASD. Specifically, 25% of the parents expressed concern in the first week of life, 37% in the first year of life, 50% between the first and second years of life, and 70% from 2 to 3 years of age [57].

The trajectories of children with ASD and TD from 1.3 to 3 years of age were analyzed and it was observed that feeding difficulties in children with ASD increased over time and were related to inappropriate behaviors during meals and food acceptance [65]. Another study compared retrospective data from 34 children with ASD (97% male) with 34 TD children (79% male) and found that children with ASD showed a significant difference in relation to the average age for the transition to drinking from a cup, starting on average at 17.7 months of age, while TD children started at 11.04 months of age. In addition, with regard to the ability to feed oneself with a fork, children with ASD started, on average, at 26.7 months of age, while TD children started earlier, at 14.8 months of age [66].

One study evaluated 65 children with ASD and 26 TD children, all male, aged between 2 and 3 years of age. In this study, it was observed that the ASD group had significantly more problems related to eating compared to the control group. These problems were classified as difficulty chewing and swallowing, sameness behavior, aggression during meals, and eating rituals, indicating an increase in food selectivity [67]. Based on the parents’ reports in questionnaires, 86 children were identified who were later diagnosed with ASD. It was observed that, as the age of the children advanced, the problems became more pronounced. At 15 months of age, parents had concerns about hearing and eating behavior; at 18 months of age, concerns about repetitive, motor, social, communication, and play behaviors were identified. At 24 months of age, differences in temperament emerged, and at around 30 months of age, there was an increase in crying and differences in bowel habits and stool characteristics. Feeding problems were more evident at 15 and 24 months of age. At 15 months of age, difficulties in establishing a feeding routine, overeating, insufficient food intake, refusal to eat certain foods, selectivity in relation to food, specific likes and dislikes, feeding difficulties, and preference to eat alone were observed. At 24 months of age, difficulties in establishing a feeding routine, overeating, insufficient food intake, refusal to eat certain foods, selectivity in relation to food, and specific likes and dislikes were observed [68]. Another study [46] evaluated 60 children with ASD (81.7% male) and 50 TD children (68% male) through the video recordings of interactions between the mother/child dyad during mealtimes. This study identified food refusal and children’s tendency to get up and walk around without a specific purpose during mealtimes. The study also observed that mothers and children with ASD with feeding difficulties seemed to have more intrusive interactions, which can impair children’s autonomous initiatives.

The analysis of data from a cohort of 348 individuals with ASD symptoms (61.8% male) between 1.5 and 4 years of age found that 53.3% of them had eating problems during this period during follow-up, including difficulties such as ‘problem eating’ and ‘eating too little’ [69]. Similarly, 4219 children with ASD (50% male) from another population-based cohort showed a robust correlation between ASD symptoms and eating difficulties at 1.5 and 3 years of age, reported as ‘does not eat well’ and ‘refuses to eat’ [60].

### 3.4. Developmental Feeding Skills in Patients with ASD Older than 3 Years of Age

Parents’ concerns about feeding children with ASD continue as they get older. It is essential to note that disruptive eating behaviors have a substantial impact on family meals [57]. The eating concerns of the parents of 16 children with ASD (75% male), aged 3 to 6 years of age, were higher (86%) in a cross-sectional study [70] which evaluated individuals with ASD up to 18 years of age. Compared to their TD peers, more parents of children with ASD reported difficulties with breastfeeding, concerns about feeding, and that their children were picky eaters and avoided certain foods. In addition, inappropriate mealtime behavior occurred regardless of where it occurred.

Evaluating 40 boys with ASD and 72 TD boys between 3 and 7 years of age revealed that age showed a negative correlation with food avoidance and eating rituals in TD children. This suggests that these behaviors decrease as the children get older. On the other hand, in the ASD group, age showed a positive correlation with food selectivity, sameness, and food rituals, indicating an increase in these behaviors with age [67].

The eating behavior of 24 children with ASD (75% males), with an average age of 4.3 years of age, was compared with that of 24 TD children of the same age and sex. The study revealed that the frequency of eating problems was 96% in the ASD group, whereas in the TD group, it was 54%. With regard to the location of meals, 96% of the children with ASD had difficulties eating in any environment, compared to 67% of the children with TD who faced this problem. Inappropriate behavior, such as leaving the table, was observed in 38% of the children with ASD, while only in 8% of the TD children. Similarly, resistance to sitting at the table was observed in 38% of the children with ASD, compared to 4% of the TD children. In addition, throwing food was identified in 33% of the children with ASD and 4% of the TD children. The occurrence of tantrums was reported in 25% of the children with ASD, but not in TD children. In addition, 62% of the children with ASD were picky eaters, in contrast to 12% of TD children who showed this dietary restriction [57].

Analyzing data from a cohort of 4155 children with ASD symptoms (50% male) at 3 years of age, a strong correlation was observed between ASD symptoms and eating problems. In addition, it was noted that higher levels of eating problems predicted a subsequent increase in ASD symptoms when the children reached 6 years of age [60].

When investigating the presence of a feeding phenotype in a sample of 185 children with ASD (72.4% male) and a control group made up of 111 TD children (46% male) between 4 and 17 years of age, it was found that 3.6% of the TD children and 18.9% of the children with ASD exhibited the characteristics of selectivity and feeding in the absence of hunger simultaneously. The ASD group showed a high frequency of ritualized behaviors and had greater difficulty managing transitions and changes in the context of eating. It is important to note that the prevalence of these characteristics was not observed in TD children. Selective eating was identified in 36.8% of the children with ASD, while 21.6% of the TD children had this problem [71].

### 3.5. Autism, Hormones, and Brain Regulation of Appetite/Satiety

Nutrition is controlled by complex physiology, such as hormones. Ghrelin, an appetite-stimulating hormone, may play a significant role in the high rates of obesity observed in individuals with ASD due to fluctuations in its levels during periods of fasting and food expectancy [72]. In contrast, leptin, predominantly secreted by adipose tissue, plays a crucial role in regulating body weight and energy balance, exerting a central influence on appetite control and energy expenditure [73]. On the other hand, adiponectin, a protein originating in adipose tissue, is involved in the control of energy metabolism, showing a significant inverse correlation with parameters associated with insulin resistance and obesity [74].

In addition to these hormones, relaxin-3, a hormonal peptide similar to insulin-7, demonstrates the capacity to influence feeding and appetite-related behavior and is considered a key molecule in these processes [75]. These observations highlight the complexity of hormonal and metabolic interactions in individuals with ASD, suggesting that changes in the levels of these hormones may contribute to the metabolic and eating characteristics frequently observed in this population.

When comparing 31 boys with ASD to 31 TD boys, both aged between 6 and 19 years of age, it was observed that serum adiponectin levels in the ASD group were significantly lower than the TD group. Additionally, a relatively high negative correlation was found between serum adiponectin levels and the Autism Diagnostic Interview-Revised domain A score (Social Interaction), indicating abnormalities in social interactions [74]. 

Adiponectin and leptin levels were compared in 40 boys with ASD to those of 40 TD boys, both groups aged between 3 and 10 years of age. The study found higher levels of leptin and significantly lower levels of adiponectin in the children with ASD compared to the control group [76]. Another case–control study [72], which involved 44 children with ASD (86.4% male) and 44 TD children (79.5% male) aged between 18 and 60 months of age, identified significantly higher plasma levels of leptin and ghrelin in the ASD group. However, there was no correlation between these levels and the severity of eating problems.

Based on data from a cohort in which participants were followed up from birth, with an average follow-up of 7.5 years of age, 53 individuals with ASD (73.6% male) and 769 TD individuals (42.5% male) were evaluated. This analysis revealed that children with ASD had higher levels of leptin in umbilical cord blood and during infancy compared to TD children. In addition, it was observed that excessive weight gain during infancy and early childhood was associated with an increased risk of developing ASD, this association being more pronounced in children with higher levels of leptin in cord blood [73]. The relationship between body weight and leptin and ghrelin levels was investigated in another study [77] which evaluated 21 participants with ASD (81% male) aged between 5 and 12 years of age divided into two groups based on body weight with 15 classified as normal weight and 6 as overweight/obese. It was observed that the overweight/obese individuals had higher leptin concentrations and faced more eating challenges compared to the normal-weight participants. However, no significant differences were found in the ghrelin levels between the two groups.

Using a sample of 169 children between 2 and 15 years of age from a cohort stratified as follows: 37 children with early-onset ASD (87% male); 33 children with regressive ASD (86% male); 50 TD children (76% male); 26 siblings of children with ASD (77% male); and 23 children with developmental disabilities (DD), this study found that children diagnosed with ASD had significantly higher plasma levels of leptin compared to the TD controls, DD, and siblings of children with ASD. Additionally, within the group of children with ASD, those with an early onset of the condition had significantly higher plasma leptin levels compared to children with regressive ASD [78].

A study evaluating the levels of relaxin-3 in 50 children with ASD (88% male) and 30 TD children (80% male), all aged between 2 and 8 years of age, found that the serum levels of this hormone were higher in children with ASD. In addition, a statistically significant positive correlation was found between the serum levels of relaxin-3 and the “desire to drink” sub-score of the Child Eating Behavior Questionnaire. At the same time, a negative correlation was observed between these serum levels and the ability to respond to satiety, associated with a reduction in food craving scores. As a result, both the ability to respond to satiety and the food craving scores were reduced [79].

The interaction between neural aspects, physiological, endocrine, and behavioral processes in the process regulation of hunger and satiety are intimately related to feeding behavior.

### 3.6. Family Influence in Child’s Nutrition

Family engagement has a key influence on children’s eating patterns, since parental feeding styles influence food preferences and eating behavior. Therefore, these aspects may impact directly and indirectly on diet quality [80]. Studies present that each parental feeding style has a different impact on the dietary intake and behavioral expression of appetitive traits [81,82]. These patterns may also influence eating problems, considering that coercive parenting styles are associated with higher levels of food fussiness and food neophobia in children [83,84].

The observation of visual cues, physical disposition, and positive or negative expressions during mealtime influences the perception of hunger and satiety signs by parents and caregivers [26]. Additionally, parental perceptions of interest or refusal of food may affect the decision-making process related to feeding. For instance, Brown and Rowan [85] observed that maternal characteristics and perceptions influenced the timing of the introduction of solid food. Data regarding parental eating style or practices in the population with ASD is limited; however, the relevance of family attitudes has been recognized and investigated in studies on the treatment of eating difficulties [86,87,88].

Concerns with feeding are commonly observed in the parents of children with ASD. In fact, more than two-thirds of parents describe a past or present concern with feeding, according to a cross-sectional study. Among the concerns identified, the most common was the limited variety of food, while being underweight and overeating were the most common issues of therapists. Insufficient nutrition, eating behavior, limited variety, and excessive intake were identified as concerns often overlooked by professionals [89].

Parental concerns regarding eating difficulties persist throughout childhood and adolescence [70]. In this context, environmental factors have an important role in the development of eating behavior, including parents’ and caregivers’ eating habits. Thorsteinsdottir et al. [90] investigated the influence of parental eating habits in children with and without neurodevelopmental disorders. Their findings indicate that children whose parents considered them fussy eaters consumed less healthy foods, such as vegetables, fruits, and milk; and had a higher frequency of consumption of unhealthy foods.

Similar results were found when investigating the associations between eating behavior and feeding practices of 76 parents and their children. The healthy eating habits of parents were significantly associated with the use of positive reinforcement and the consumption of fruits and vegetables by children [91]. Thus, the parental observation of feeding cues and eating behavior has a significant impact on the development of eating habits and self-regulation in children [92].

Parental habits and behavior influence children’s eating and feeding problems and can also have an impact on family routines and practices. The study by Gent et al. [93] concluded that the parents of children diagnosed with ASD and feeding difficulties have a greater impact on their daily lives and a higher level of worry. In addition, Zlomke et al. [94] observed that anxiety and maternal strategies during meals influence the eating behavior of children with ASD and may contribute to the persistence of eating problems. This highlights the importance of parental guidance on the perception of the signs of feeding problems, and on the cooperation to work with these issues in an appropriate manner.

A complete feeding evaluation is crucially important in relation to the development of a child. It is extremely important to consider all the aspects of feeding, whether biological or behavioral, so that appropriate intervention can be recommended for the patient and the family. Specifically for patients with ASD, as discussed above, it is important to consider the influence of hormones, such as leptin and ghrelin, which could influence the brain’s regulation of appetite and satiety. Additionally, parent’s feeding behavior should also be evaluated for the influence they can have on their children’s diet. All these variables have an effect on feeding skills and food intake and need to be considered in the intervention plan for these patients.

### 3.7. From Literature: Series of Cases

To illustrate the developmental course of children with atypical feeding behaviors in children with ASD, several case reports are summarized below describing such children.

According to pediatric records, the past history of a girl with ASD and severe food selectivity was collected at 28 months of age. Physical development milestones were reached normally; at 10 months, she uttered babbling words, but lost her language at 12 months. Prior to this, the girl was curious about food; at 8 months, she accepted new foods, and at 10 months, she was fed the family’s own mashed food. At 15 months, she developed food selectivity, showing total refusal of food. At 20 months, she began treatment with an Occupational Therapist for her eating difficulties for a period of 6 months without noticeable progress. At 23 months she began to show outbursts of anger, severe fine motor and visuo-motor delays, decreased bilateral strength, and resistance to all therapy tasks. At 28 months, her diet was restricted, with less than 10 foods accepted, consisting mainly of breast milk, bananas, chicken nuggets, and fruit and vegetable smoothies. The girl’s behavior included refusing to eat unfamiliar food, screaming, crying, and raging, even at food eaten by other people. If unknown food entered her mouth, she choked until she vomited [95].

A prospective case series [96] followed five children from the age of six months of age who were subsequently diagnosed with ASD at 36 months of age, and identified eating issues in the assessments. At 10 months, one boy showed resistance to being fed. Another boy at 12 months showed a refusal to eat foods with a non-soft consistency. Two girls at 12 months showed opposite behaviors, with one exhibiting limited food preferences, while the other consumed a variety of foods. Another girl at 18 months showed strong food preferences, exemplified by her aversion to meat and her acceptance of fish only when pureed.

A series of case studies reported on the eating behavior history of four children with ASD, with no associated medical issues. All the participants faced challenges in the transition from weaning to solid food. One boy was diagnosed with ASD at 3.1 years old and had refused solid food since he was two years of age. By 4 years of age, he was consuming only three types of food and was over-consuming milk to the point of vomiting. There was a slight improvement in food selectivity over a period of 5 years. Another boy diagnosed with ASD at 3.8 years of age had problems with breastfeeding during the first months of life and later showed distress and refusal if infant milk was delayed. He showed no enjoyment of any food. By 4 years of age, he only consumed one type of food and refused to eat. Another boy diagnosed with ASD at 3.11 years of age refused solids, showed no interest in mealtimes, and was passive about eating. A girl diagnosed with ASD at 5.4 years of age had stable and unchanged food restrictions at 7 years of age [97].

Case reports are not considered scientific studies with a gold standard of methodology. Among some questionable points, it is important to remember that these studies do not have control groups, for example. However, they can bring specificities to the description of patients and point out characteristics that are often not common to the vast majority of patients.

## 4. Conclusions

Developmental milestones are important aspects to be monitored during child development; they involve social, motor, and cognitive aspects. Eating, despite being a constant and daily action throughout life, is often indicated as something instinctive. Eating is a necessary skill related to developmental milestones; however, signs of hunger and satiety can also be classified according to age and can be used to detect atypical development in feeding. These signs, within the typical course of child development, contribute to understanding the sensations of hunger and satiety, as well as help to build appropriate eating habits. A high percentage of patients with ASD have eating difficulties and these signs can be manifested in the first year of life. Eating symptoms are not currently included in the diagnostic criteria for ASD; however, they can serve as a warning sign for parents and caregivers. The manifestation of these symptoms does not mean that the child has or will develop ASD; however, it can be a warning sign that contributes to the search for a specialist by families, as well as alerting professionals in the area to closely monitor their patients for these signs and symptoms.

Although this study provides valuable insights into the early feeding signs in ASD, there are several avenues for future research that could build upon our findings. Firstly, prospective studies in this field would be beneficial to extend this research. Additionally, specific tools can be developed in order to examine these factors in greater depth.

## Figures and Tables

**Table 1:** Signs of hunger and satiety and warning signs in child development.

Age	Hunger and Satiation Signals during Development	Alert Signals Related to Feeding Development
≤3 months	(H) Open the mouth looking/search for a mother’s nipple or bottle (H) Easily suck and swallow during feeding (H) Movement of the tongue back and forward to suck (H) Signs of hunger about 8 to 12 times in 24 h (H) Posture of attention to feed moment (H)/(H/S) Sucking reflux (S) Release of the breast/bottle when satisfied	Oral and motor breastfeeding difficulties Lack of eye contact while breastfeeding Absence or low sucking reflex Loses a lot of breastmilk or formula out of the side of the mouth while feeding
3–6 months	(H) Clicking tongue (H) Grumble or cry (H)/(H/S) Hands over mouth	Deficits with mouth-opening anticipation during feeding Low acceptance or resistance to change in feeding routine Low acceptance and resistance to changes in infant formula (e.g., temperature, viscosity) Does not bring hands to their mouth
6–9 months	(H) Demonstrates interest in food (H) Active search for food (H) Leans forward to get closer to the breast or spoon (H) Holds the hand of the person offering food to “speed up” (H) Available/encouraged to try new foods (S) Turns the head and body (S) Loses interest in eating (S) Pushes the person’s hand away (S) Closes the mouth (S) Decreased activity levels or tone muscles when satiety (S) Verbal and non-verbal negative expressions	Low acceptance or resistance to change in utensils (e.g., feeding bottle, spoon) Difficulty in introducing solid foods Difficulty in drinking water Resistance to trying new foods Difficulty in making the transition from feeding bottle to glass Has a hard time getting things into their mouth
9–12 months	(H/S) Initial skills in using a spoon or fork (H/S) Reacts strongly to new smells and tastes (H) Points to food (S) Eats more slowly (S) Pushes food away or closes mouth	Presents attachment to the utensils used, demonstrating irritability when changing things Fine motor difficulty Reduction in the number of foods usually consumed
1–2 years	(H/S) Eats a greater variety of foods (H/S) Starting to learn how to eat on their own, more independent (H/S) Can hold and drink from a cup (H/S) Chews with full jaw movements (H/S) Uses utensils with some spills (H) Combines words and gestures to express the desire for food (H) Takes the caregiver to the food (S) Shakes head, says does not want it (S) Leaves the table (S) Play with food (S) Throws the food away	Inappropriate behavior during mealtime (not staying at the table, shouting, crying) Rituals related to mealtime Difficulties with chewing/swallowing A small repertoire of foods consumed Lack of interest in food Lower intake than usual for no apparent reason Persistent interest in mouthing things (e.g., tags)
3 years	(H/S) Understands social events related to meals (e.g., birthday cake for a birthday party)	Can develop strategies to avoid certain foods Make their own rituals related to mealtime Difficulty eating in unusual environments High frequency of eating during the day even when not hungry

(H) signal of hunger; (S) signal of satiety; (H/S) milestone that could be related to feed or signal that can be applied to both hunger/satiety [17,18].

**Table 2:** Instruments to assess feeding difficulties *.

Instrument	Purpose	Non-ASD	ASD	Parent’s Perceptions
About Your Child’s Eating (AYCE) [27]	It assesses the eating relationship between parents and children (8 to 16 years old), taking into account the child’s resistance to eating, the creation of a positive environment during mealtimes, and possible parental aversions during mealtimes.	X		X
Adult Picky Eating Questionnaire [28]	To assess picky eating behaviors and attitudes in adults.	X		
Aut-Eat Questionnaire [29]	Captures the range of eating problems and patterns in ASD.		X	
Baby Eating Behavior Questionnaire (BEBQ) [30]	Measures appetite characteristics during the period of exclusive milk feeding (before the introduction of solid foods).	X		
Behavioral Pediatrics Feeding Assessment Scale (BPFAS) [31]	Assesses a variety of problematic and desirable eating behaviors, as well as children’s behavior during meals, oral motor skills, and parents’ feelings and strategies.		X	
Brief Autism Mealtime Behavior Inventory (BAMBI) [32]	Evaluate feeding problems in children with special needs, specifically those with ASD and other developmental disabilities.			X
Caregiver’s Feeding Styles Questionnaire [33]	Measures demandingness and responsiveness with regard to child feeding.	X		X
Children’s Eating Behavior Inventory (CEBI) [34]	Evaluate eating problems and eating behavior in children, taking into account factors related to the child, the parents, and the family system.	X	X	X
Child Eating Behavior Questionnaire (CEBQ) [35]	Evaluate food approach behaviors.	X	X	
Children’s Eating Behaviour Questionnaire for toddlers (CEBQ-T) [36]	Assess the appetitive traits of toddlers (1 to 3 years).	X		
Feeding-Swallowing Impact Survey (FS-IS) [37]	To assess the impact of feeding on the caregiver, including in relation to feeding activities.		X	X
Food Behavior Checklist [38]	Measure parents’ eating behaviors.	X		X
Infant and Young Child Feeding Questionnaire for the Assessment of Knowledge, Attitudes and Practices among Child Care Providers (ICFQ-CCPQ) [39]	Assess the knowledge, attitudes, and practices of childcare providers in relation to infant and young child feeding. It is specifically adapted for childcare providers, who are on the front line in childcare.	X		X
Healthy Kids Questionnaire [40]	To assess the health and well-being of children and adolescents, it covers aspects such as eating habits.	X		
My Child at Meal Time Questionnaire (MCMT) [41]	Measure feeding styles with parents of preschool-aged children.	X		X
Mealtime Behavior Questionnaire (MBQ) [42]	Assess types of eating difficulties in young children (2 to 6 years), focusing exclusively on the frequency of the child’s eating behaviors during mealtimes.	X	X	X
Montreal Children’s Hospital Feeding Scale (MCH-FS) [43]	To identify and measure the severity of behavioral and feeding difficulties, validated for children aged 1 to 6 with ASD.	X	X	X
Pedi-EAT [44]	Evaluate problematic eating behaviors in children, with a focus on how food is offered, late development of skills, and reflux. Can potentially be used in children with neurodevelopmental delays, as long as the age group and specific characteristics of each child are taken into account.	X		X
Responsiveness to Child Feeding Cues (RCFCS) [45]	Assess the quality of observed feeding interactions during infancy and toddlerhood (up to 2 years).	X		
Scale for the Assessment of Feeding Interaction (SVIA) [46]	The Italian adaptation of the Feeding Scale–Scale for the Assessment of Feeding Interaction, evaluates interactions between parents and children during feeding sessions.		X	
Screening Tool of Feeding Problems applied to children (STEP-CHILD) [47]	Identify behavioral problems associated with eating in both the child and the parents.		X	X
Yale Food Addiction Scale for Children [48]	Evaluating food in the absence of hunger.		X	

* Protocols mentioned in studies included in this review.

## Data Availability

No new data were created or analyzed during this study. Data sharing is not applicable to this article.
